# Analyzing the Number of Common Integration Sites of Viral Vectors – New Methods and Computer Programs

**DOI:** 10.1371/journal.pone.0024247

**Published:** 2011-10-14

**Authors:** Ulrich Abel, Annette Deichmann, Ali Nowrouzi, Richard Gabriel, Cynthia C. Bartholomae, Hanno Glimm, Christof von Kalle, Manfred Schmidt

**Affiliations:** 1 Department of Medical Biometry, University of Heidelberg, Heidelberg, Germany; 2 Department of Translational Oncology, National Center for Tumor Diseases (NCT) and German Cancer Research Center (DKFZ), Heidelberg, Germany; National Institute of Dental and Craniofacial Research, United States of America

## Abstract

Vectors based on γ-retroviruses or lentiviruses have been shown to stably express therapeutical transgenes and effectively cure different hematological diseases. Molecular follow up of the insertional repertoire of gene corrected cells in patients and preclinical animal models revealed different integration preferences in the host genome including clusters of integrations in small genomic areas (CIS; common integrations sites). In the majority, these CIS were found in or near genes, with the potential to influence the clonal fate of the affected cell. To determine whether the observed degree of clustering is statistically compatible with an assumed standard model of spatial distribution of integrants, we have developed various methods and computer programs for γ-retroviral and lentiviral integration site distribution. In particular, we have devised and implemented mathematical and statistical approaches for comparing two experimental samples with different numbers of integration sites with respect to the propensity to form CIS as well as for the analysis of coincidences of integration sites obtained from different blood compartments. The programs and statistical tools described here are available as workspaces in R code and allow the fast detection of excessive clustering of integration sites from any retrovirally transduced sample and thus contribute to the assessment of potential treatment-related risks in preclinical and clinical retroviral gene therapy studies.

## Introduction

Various clinical gene therapy trials have been carried out demonstrating a clear benefit for many of the treated patients [1], [2], [3], [4], [5]. In preclinical studies and in some of the clinical trials using viral vectors, various side-effects due to vector integration in the genome have been observed, ranging from immortalization [6] to clonal dominance [4], [7], [8], [9] and even oncogenesis [10], [11], [12], [13], [14], [15], [16], [17], [18]. Integration site (IS) analysis via linear amplification-mediated PCR and high-throughput sequencing [19], [20], [21] has proven to be a highly efficient technology for uncovering IS distribution in a large scale and for integration induced effects on the surrounding genomic DNA regions. Of particular interest is the formation of clusters of integrations, termed common integration sites (CIS), as an indicator for clone selection [22], [23], [24]. To evaluate if the observed clustering may have occurred by chance it is necessary to compare the experimental results with those to be expected under an assumed model distribution. Because it is known that γ-retroviruses show a different IS pattern than lentiviruses [25], [26] we developed specific tools for these IS distributions.

Here, we describe methods and computer programs for the statistical analysis of the number of CIS as well as the number of IS involved in CIS. All computer programs referred to in the sequel were written in R code (cran.r-project.org). Technical details are provided in the Supporting Documents.

## Methods

### Definitions, abbreviations and conventions

The following terminology will be used: A CIS of order n is defined as an n-tuple of IS such that the maximum distance between the elements is no greater than a fixed bound d_n_, the window size used for defining the CIS. While in our examples with relatively small sample sizes we chose the window sizes for CIS definition (d_2_ = 30 kb, d_3_ = 50 kb, d_4_ = 100 kb, and d_n_ = 200 kb, for n>4) to be identical to those used in earlier investigations [22], [24], our methods and programs allow for an arbitrary choice of d_n_, a feature that may be useful with increasing sample sizes after high throughput sequencing. As for alternative definitions of CIS proposed in the literature [27], [28], the last section of this paper will briefly analyze how our approach relates to these developments.

Notation used in the sequel:

is   number of observed IS in the part of the genome under study

cis_n_   number of CIS of order n

iscis_n_   number of IS involved in CIS of order n

E(X)   expected value of the random variable X

g   length of the genome or the part of the genome under study

TSS   transcriptional start sites

n_TSS_   number of TSS in the particular part of the genome under study

I_TSS_   interval(s) around a TSS possibly affected by preferential insertion of γ-retroviral vectors (the interval is assumed to be symmetric around the TSS)

w   halfwidth of the interval(s) I_TSS_

p_TSS_   proportion of IS allocated to the I_TSS_

p_pref_   proportion of the TSS affected by the preference

G,H   gene coding region and its complement (resp.) in the particular part of the genome under study

q_G_,q_H_   proportion of IS assumed to insert into gene coding regions and the complement (resp).

### General aspects

This paper is concerned primarily with the *number* of CIS (or IS involved in CIS) of a given order n. Generally, the analysis is based on an assumed spatial distribution, f_IS_, of the IS. In statistical terms, this represents a null hypothesis. Expected values of cis_n_ or iscis_n_ under H_0_ are calculated, and the observed numbers are compared with their statistical distribution f_cis,n_ and f_iscis,n_ (resp.) under H_0_, yielding p-values.

### Two approaches were adopted (the first one applicable only to the number of CIS):

1. Mathematical formulae for the expected value along with assumptions regarding the distributional form of f_cis,n_. We assumed a Poisson distribution, which is an approximation to (and a limiting distribution of) the binomial in case of rare events. Thus, the Poisson distribution does not concern the IS but the number of CIS of order n. The approximation may be used if the probability of a random IS to be part of a CIS is small (<5%).
2. A more general and comprehensive approach relying on computer simulations of f_IS_. In contrast to approach (1) this allows to take into account the spatial structure of the genes or the TSS. With computer simulations, no parametric model (like the Poisson distribution) for the distribution of the CIS is required.

Some explanations are in order to understand the scope of the analyses. If a fixed distribution f_IS_ is assumed then the analysis will merely yield a conclusion about whether or not the observed number of CIS of order n, cis_n_, is compatible with this assumption (compatibility being measured by the p-value). A small p-value is indicative that the degree of clustering is stronger than implied by the model.

As an alternative, a *family* of IS distributions may be assumed, the members of which differ in the values of one or more parameters. Thus, e.g, retroviral distributions with preference for the neighborhood of TSS may differ in the assumed width of this neighborhood and in the degree of the preference. Then two types of questions may be asked: (i) whether cis_n_ is compatible with certain given values of the parameter(s); and (ii) how the parameter(s) must be chosen so as to be statistically compatible with the observed number cis_n_, or even so that the expected number of CIS is *equal* to cis_n_.

Finally, in some of the methods developed for comparing the number of CIS observed in two studies with different numbers of IS, the distributional assumption for the IS is not directly used for calculating expected values, but is rather treated as a nuisance parameter which determines the (necessary) adjustment of the results of the comparison.

As for p-values, in many of our computer programs the user can select the direction of the statistical tests, namely, one-sided (upper tail or lower tail) or two-sided testing. Whenever H_0_ stipulates a uniform distribution, however, only one-sided testing is appropriate.

Whenever a p-value, p_sim_, is based on computer simulations, it is only an estimate of the true p-value p (which is a probability). If, e.g., the test statistic is given by the number of observed CIS of order n, the (one-sided) p_sim_ is defined by the ratio of the number of simulation runs resulting in at least cis_n_ (i.e., the number observed in the experimental sample) CIS of order n, to the total number of simulation runs, nsim. As pointed out by Li et al. [29], it then is advisable to calculate upper confidence bounds for p, based on p_sim_. This is easy to accomplish, given that p_sim_ follows a binomial distribution B(nsim,p). In our programs, whenever analyses are based on simulations, exact one-sided, test-based 95% upper confidence bounds (Clopper-Pearson bounds) for the true p-values regarding the overall (i.e., not the chromosome-specific) results are calculated.

Another aspect is the multiplicity of tests. Most analyses generate more than one p-value, due to the fact that different orders of CIS are analyzed and/or different distributional assumptions (corresponding to different null hypotheses) are made. Hence, in some situations issues of multiple testing arise. There are numerous methodological strategies for dealing with multiple testing, see, e.g., Hsu [30] for a survey of the issues and approaches. The methods and programs described here leave the choice of how to adjust for multiplicity to the user, and, therefore, these questions will not be addressed further in this manuscript. The reader is strongly advised to formulate the testing strategies, and thus the use and interpretation of the p-values, prior to the data analysis.

## Results

### Modeling a uniform distribution of the IS

While it is known that γ-retroviruses do not show a uniform integration pattern, analyses of this type may be of interest when it comes to lentiviruses, see below.

In Abel et al. [31] a mathematical framework was derived for the calculation of expected values E(cis_n_) of CIS of order n (n = 2,3,4) under the null hypothesis that the IS are uniformly distributed. In principle, it is possible to derive formulae for orders n>4 using the recursive approach given in Abel et al. [31]. The k-th order requires a formula for 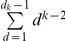, which can be obtained following the line set out in Heuser [32], page 130.

The resulting formula for CIS of order n = 5 is given in the Supporting Document Text S1.

The formulae were implemented in elementary programs *cis* and cisv yielding expected values and p-values based on a Poisson distribution of the number cis_i_ of order i (i≤5). Note that in most practical applications E_5_≪1 so that, assuming a Poisson distribution for cis_n_, the p-value of a single observed CIS of order 5, is≈E_n_ and thus≪1, as well. Since E_n_<E_n−1_, the observation of at least one CIS of order n implies a p-value of p<E_5_≪1, an upper bound that is satisfactory in most cases. Hence a formula for E_n_, n>5, is rarely needed.

Generally, the approximations involved in the formulae are excellent. However, while the formula-based approach allows a very quick, rough orientation, in many situations computer simulations will be more satisfactory. First, the formulae may be dubious if g is not considerable larger than the window size d_n_. Second, no formulae have been derived for orders >5. And third, no formulae are available for the number of IS involved in overlapping CIS. It is only when the CIS of order n can be assumed to be extremely sparse, so that overlaps can be neglected, that this number is approximately equal to cis_n_*n.

### Modeling a more general γ-retroviral distribution of the IS

The term γ-retroviral distribution will be used to designate a distribution of the insertions which assumes that insertions occur preferentially in the vicinity of the TSS, but are uniformly distributed in the remainder of the genome [26]. A distribution of this type was used in the analyses carried out by Wu et al [33].

Mathematically, the γ-retroviral distribution is a parametric class of distributions, the parameters being

- the halfwidth w of the intervals I_TSS_
- the proportion p_TSS_
- the proportion p_pref_.

(see above). As is easy to see, the uniform distribution is a special case of this class.

To obtain mathematical formulae, it must be assumed that the preferential allocation of IS expressed by p_TSS_ and p_pref_ is independent on the particular location of the TSS.

In Abel et al. [31] general formulae were derived for calculating the expected number of CIS of order 2, given the values of the parameters mentioned above, and solutions of these equations for the case w = 5 kb were presented. In the Supporting Document Text S2 the solutions are given in a more general form allowing for arbitrary w, and including a slight correction. It is important to note that, as long as w<d_2_/2, the expected values do not depend on the spatial distribution of the IS inside of the I_TSS_, as proven in the Supporting Document Text S2.

Again, this approach (made available in the program *cisretro*) is useful for a quick approximate analysis using hypothetical values for the parameters, in particular p_pref_. Note that p_TSS_ can be estimated from IS and TSS data (as the proportion of IS lying in the union of the I_TSS_), and whenever an estimate is available it may be used in place of a hypothetical value.

#### Example 1

For the human genome, Wu et al. [33], using computer simulations based on is = 1,200, p_TSS_ = 25%, p_pref_ = 5%, w = 5 (kb), obtained E(cis_2_)≈55. A recalculation by means of the mathematical formulae described above (with n_TSS_ = 20,484, g = 312,000,000 kb) yielded E(cis_2_) = 56.1.

However, the assumption underlying the formulae, namely that CIS arising from IS located in two different (e.g. overlapping) I_TSS_ are negligible, may be problematic. As can be easily calculated, this approximation is, indeed, justified (with w = 5 kb) if the TSS can be assumed to be uniformly distributed. In reality, however, the distribution of the TSS in the genome is far from uniform, but rather shows a marked clustering, which then, by virtue of the preferential allocation of IS, may increase the expected number of CIS beyond the values implied by the formulas if a high percentage of IS are located in the I_TSS_.

This observation is highlighted by the positions of the first 15 TSS on chromosome 1:
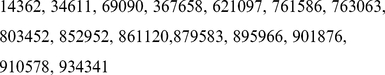
While these 15 TSS have a span of almost 1,000 kb, no less than 10 of them lie in an interval of 250 kb (between position 750 and 1,000 kb), and in several cases the I_TSS_ with w = 5 kb will even overlap.

In other words, in order to perform a well-founded analysis for γ-retroviral insertions, computer simulations are needed that take into account the *exact* positions of the TSS (see below).

### Modeling a lentiviral distribution of the IS

Lentiviruses are known to insert preferentially into the gene coding regions [25]. Conditional on this preponderance their IS are thought to be uniformly distributed both in the gene coding regions G and their complement H.

If this assumption holds true, then statistical analyses - not taking into account the exact position and length of every single gene - can be carried out by applying the methods developed for uniform distributions separately to the gene coding regions and their complement.

This approach was implemented in the program *cislenti*. The program yields formula-based expected values for cis_n_, n = 2,…,5, as well as p-values derived from Poisson distributions with these expected values, evaluated separately for G and H, as well as for G∪H.

Again, this formula based approach is mainly meant for quick hypothetical model-based calculations (“scenarios”).

#### Example 2

We consider a data set of lentiviral IS in dividing mouse cells (SC-1 mouse fibroblasts and hematopoietic progenitor cells), analyzed by our group [34].

The integration site analysis yielded 611 IS, forming a total of 33 CIS of order 2. Using the program *lenti* with the parameters pertaining to the mouse (g = 2654855048 b, length of G = 939587421 b), it was found that under the null hypothesis of a lentiviral distribution the expected value E(cis_2_) ranged from 4.2 (for q_G_ = 35.4%, a value corresponding to a uniform distribution of the IS and also obtained using the program *cis*) and 11.9, attained for q_G_ = 100%. Equating q_G_ to the sample value of 77.1% yielded E(cis_2_) = 7.4. Regardless of the true value of q_G_, the observed value of cis_2_ was significantly higher (p<10^−6^) than the expected value.

A caveat similar to that made for γ-retroviral analysis also applies to lentiviruses: The formula-based analysis, which treats the gene-coding regions and their complement as connected intervals, may be questionable if the number of IS is high so that many CIS are formed by combinations of IS from G and H. A more appropriate analysis, taking into account the exact structure of the regions is provided by the programs described in the next paragraph.

### Simulation-based CIS analysis using IS location data

The basic methods and programs described above are cornerstones for more comprehensive analyses of IS data. Given a data set of IS locations, the analysis of CIS comprises at least the following steps:

1. Determine the number of CIS of order 2,3….(In our programs the maximum order analyzed was n = 30.)
2. Determine the location and number of IS involved in CIS of order 2, 3…
3. Compare these numbers with the expected values under a uniform distribution, the γ-retroviral distribution with preference for I_TSS_, or a lentiviral distribution, as described above. I.e., these distributions are the null hypothesis H_0_ to which the p-values refer.

All steps are performed both for each chromosome separately and genome-wide. For each distribution two separate methods (denoted by the suffix c and u, resp.) were implemented representing a conditioning of the analysis on the observed numbers of IS on the chromosomes and the observed values of the model parameters, and an analysis without this conditioning, respectively. Additional technical remarks can be found in Supporting Document Text S3.

The unconditional versions were mainly intended to test different hypothetical models. Therefore, the assumed model parameters (e.g., in case of lentiviral distributions: the proportion q_G_ of IS inserting in gene coding regions; in case of γ-retroviral distributions: the parameters p_TSS_, p_pref_) have to be furnished as program input. For chromosomes and those model features which are observable (this is not the case for p_pref_, for which no straightforward method of estimation is available) the unconditional version of the programs then yields p-values of the chisquared goodness-of-fit test for the IS. E.g., in case of the uniform distribution, it is tested whether the observed numbers of IS on the chromosomes differ from those expected under H_0_ (which are proportional to the length of the chromosomes). In case of γ-retroviral and lentiviral models, additional goodness-of-fit tests are carried out regarding the assumed values of the model parameters p_TSS_ and q_G_, respectively.

The programs providing a conditional analysis are conditional both on the observed number of IS on the chromosomes and on the observable model parameters (p_TSS_ in case of γ-retroviruses and q_G_ in case of lentiviruses).

Thus, in all, the package comprises 6 programs carrying out steps 1 to 3: *CISUNIFc, CISUNIFu, CISRETROc, CISRETROu, CISLENTIc, CISLENTIu*.

### Some details may be of interest:

**Figure 1:**
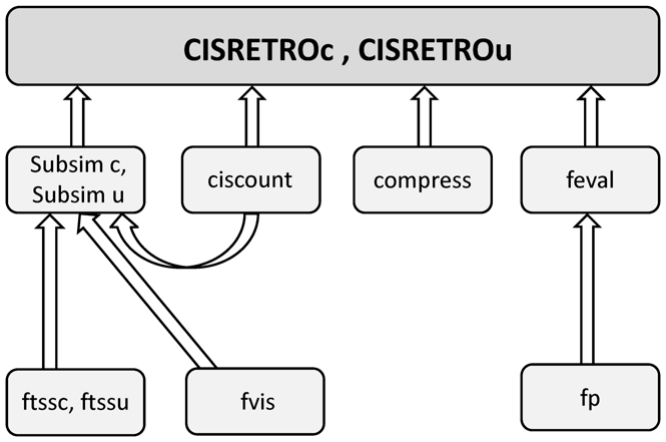
Structure of CISRETROc, CISRETROu. The programs CISRETROc and CISRETROu give the expected numbers and p-values of CIS and IS involved in CIS based on a γ-retroviral IS distribution using Monte-Carlo methods. 7 subprograms work together to produce the results. fp: calculates p-values based on the simulated distribution of results; fvis: generates uniformly distributed IS locations; ftssc, ftssu: generate randomly distributed IS in the I_TSS_; feval: carries out the statistical analysis; compress: compresses highly disconnected genomic regions produced when discarding the I_TSS_; ciscount: counts the CIS; Subsim_c, Subsim_u: carry out the simulations and count the CIS for each simulation run.

- All analyses require the specification of the species under investigation (rat, mouse, human). This determines the number and length of the chromosomes used in the analysis.
- The γ-retroviral analysis (*CISRETROc, CISRETROu*) makes use of a global matrix containing the positions of all TSS for each chromosome (for humans, this amounts to a matrix with about 20,000 rows). The main challenge of the analysis consisted in producing uniform distributions in the complement of the I_TSS_, which can be visualized as a continuum with about 20,000 holes of identical size, many of which overlap.
  For simplicity, the retroviral analysis assumes a uniform distribution of the IS within the I_TSS_. As has been mentioned above, this special choice will hardly affect the number of CIS, given that the distribution inside of the I_TSS_ plays a role only for CIS arising from overlapping I_TSS_. Also, as before, it is assumed that the preferential allocation of IS is independent on the particular location of the TSS so that random samples of the TSS can be drawn when modeling H_0_. The structure of the programs *CISRETROc* and *CISRETROu* is shown in Figure 1.
- The analysis of lentiviruses requires the exact positions of all genes on the chromosomes (stored as a global matrix in the R workspace).
- No separate counting of CIS is done for the union of the I_TSS_ in case of γ-retroviruses and for gene-coding regions or their complement in case of lentiviruses, because, as mentioned above, these regions are highly disconnected and composed of subintervals many of which are smaller than the defining window sizes for CIS.

#### Example 3: γ-retroviral vectors

As mentioned above, because of the overlap of the I_TSS_ (as is the case on human chromosome 1) the formula-based approach may be unsatisfactory when dealing with a large number of IS which are heavily concentrated in the I_TSS_. To support this claim we consider an example of 319 IS on chromosome 1 (a value found in one of our studies), and assume the extreme case that p_TSS_ = 1. If p_pref_ = 1 the mean value of CIS of order 2 obtained in 10,000 simulation runs taking into account the length of the first chromosome (249,250,621 bp) and the exact location of the 2,135 TSS on this chromosome, was 50.4, compared to a formula-based expected value of 23.7.

#### Example 4: Lentiviral vectors

We applied the program *CISLENTIc* to the data set described in *Example 2*. The mean value of cis_2_ obtained in 10,000 simulation runs was 6.75, with an empirical p-value of 0, yielding an upper 95% confidence bound for the true p-value of 0.0003. That the simulations result in a slightly lower expected value than the formula may be due to the fact that the formula treats the gene-coding regions as a connected interval when in reality they are highly disconnected. We also used the program *CISLENTIu*, in which the number of IS allocated to gene coding regions of each chromosome are proportional to the length of these regions (and not, as in CISLENTIc, to the number actually observed). We alternatively set q_G_ = 77.1% ( = the observed value) or q_G_ = 75%, the latter value being equally statistically compatible with the observed proportion of IS in gene-coding regions, as judged by a non significant result of the goodness-of-fit test for the model. This yielded even lower mean values of 6.3 and 6.1 (resp.) CIS of order 2, respectively (10,000 runs each).

### Comparing results from two vector integration studies

In many experiments it is necessary to compare the results (locations of vector integrations) from two vector integration studies e.g. when the IS profile of two different vectors used in clinical trials have to be determined. One aspect of interest is the inherent propensity of the IS of these vectors to form CIS. Often the patient material that can be used for integration site analysis is limited so that it is not possible to get a comparable amount of DNA. Usually this implies that the numbers is_1_, is_2_ of observed IS in the two samples will be different. The challenge with such an unbalanced comparison is that the number of IS itself affects the expected number of CIS. Even with random uniform allocation this dependency is strong. Thus the challenge arises how to eliminate the influence of the sample sizes of the IS on the comparison of the CIS.

We have taken two different approaches to this challenge. The first applies to the number of CIS only. It has a firmer theoretical foundation but depends explicitly on some assumptions regarding the distribution of the IS. The methods exploits the general fact that if X_1_ and X_2_ have Poisson distributions with parameters (i.e., expected values) λ_1_ and λ_2_, respectively, then the difference X_1_–X_2_ follows a Skellam distribution with parameters λ_1_,λ_2_. (The Skellam distribution is available as a CRAN package in R.) In the applications the true expected values λ_1_,λ_2_. are unknown. However, they can be calculated (either from a formula or from simulations) if a particular model for the distribution of the IS is assumed.

For the γ-retroviral model proposed by Wu et al. [33] one can use the formulae given in the Supporting Document Text S2 for calculating expected values. Thus, we have a parametric model with the structural parameters p_TSS_ and p_pref_. As mentioned before, this approach can be considered approximately valid if the values of p_TSS_ and p_pref_ are not too extreme. Here, it is assumed that 0.1≤p_TSS_≤0.5 and 0.1≤p_pref_≤1. For each pair of structural parameters, a p-value can be calculated from the Skellam distribution. In this analysis, p_TSS_ and p_pref_ are nuisance parameters. To eliminate these parameters we follow the approach originally proposed by Barnard [35] for significance tests for 2×2 tables, in which the p-value is taken as the supremum of the p-values over the admissible region for the nuisance parameters. This method is implemented in the program *compsk_retro* which, based on the observed difference of CIS of order 2 in the two samples, calculates p-values for a two-dimensional grid of (p_TSS_, p_pref_) with step width of 0.1 and 0.2, resp., and determines the maximum of these p-values. Note, however, that the formula-based method described above is limited to CIS of order 2, and it cannot be applied for the number or proportion of IS involved in CIS.

The second approach (programs *comp1*, *comp2*) is less limited in scope and does not have any explicit distributional assumptions, but is somewhat heuristic. It is based on a Monte-Carlo method which adjusts for the differences in the number of integration sites.

The method has been implemented for the number/proportion of IS involved in CIS (for which no Poisson distribution can be assumed). Briefly, it proceeds as follows: Let IS_1_ and IS_2_ be the samples of is_1_ and is_2_ integration sites, respectively, and assume first that is_1_≫is_2_. Random samples of size is_2_ are drawn repeatedly (say, nsamp times) without replacement from IS_1_, and for each of these samples the numbers of IS in CIS of different orders are counted. This yields simulated distributions of these numbers, with which the observed numbers of CIS in IS_2_ are then compared to obtain empirical p-values.

If is_1_≈is_2_ this method is unfeasible, however, because all random samples will become highly similar. A variant of the method can then be tried using nsamp random subsamples of identical size ≪min(is_1_, is_2_) from *both* IS_1_ and IS_2_. Each subsample from IS_1_,IS_2_ then yields a number of IS in CIS of order n, and these resulting values (x_1_,…,x_nsamp_), (y_1_,…,y_nsamp_) pertaining to IS_1_ and IS_2_, respectively, can then be compared using a suitable test (we use the Wilcoxon rank sum test). The whole procedure should be repeated several times to obtain more reliable p-values (see below).

We emphasize that - exceptionally - drawing *with* replacement, i.e. bootstrapping, is not applicable in this context. Generally, the bootstrap is not a suitable tool for investigating questions that have to do with the spatial clustering of data points. The reason is that bootstrap samples will produce a distance of exactly 0, if the same data point is drawn twice. I.e., the bootstrap sample will contain many clusters even if the original distribution is uniform.

At first glance, since the samples of IS which are the basis for the calculation of p-values are of identical size and only the sample distribution is used, the comparisons involved in this method appear to be neither affected by the differences in the sample sizes is_1_ and is_2_, nor to depend on distributional assumptions for the IS. However, as extensive simulation studies have shown, this is not true. There is a dependence on various parameters conveyed by an inflation of the type I error, which, incidentally, is generally much higher in case of variant 2 than variant 1. This inflation is due to the fact that drawing (without replacement) from the samples of IS is not the same as drawing repeatedly from the theoretical parent distribution of the IS.

The inflation of the type-I error means that for every concrete data analysis a simulation study must to be carried out in order to determine how the nominal α-level needs to be adjusted.

#### Example 5

To illustrate the application of the method and the α-adjustment, consider two real samples of 2,289 vs 1,152 γ-retroviral IS [Deichmann et al., unpublished results]. The samples contained 2,078 vs 161 CIS of order 2, which comprised 823 vs 236 (i.e.35.9% vs 20.5%) of the IS. The empirical p-value for IS in CIS of order 2 produced by variant 1 (10,000 runs) was p = 0.0038, whereas variant 2 (10 pairs of samples of size is_2_/2 = 576, 1,000 repetitions) yielded p = 0.0009. The simulation study analyzing the type-I error for this situation and assuming a uniform distribution of the IS resulted in estimated real α levels of 10.2% and 21.0% for variant 1 and 2 (resp.). Also, it was found that the nominal significance level would have needed to be lowered to 2.2% and 0.39%, respectively, to result in a real type-I-error of α = 5%. Note, that the results of the comparison remained highly significant even after the adjustment.

### Coincidences of vector integration sites in different cell types

In a recently carried out hematopoietic stem cell gene therapy of ALD in two patients [36], the insertion sites of the lentiviral vectors in purified lymphoid CD3^+^ and CD19^+^ cells were compared, among others, to those found in CD14^+^ and CD15^+^ myeloid cells to determine whether multipotent early hematopoietic progenitors had been transduced. If the number of observed coincidences exceeds that to be expected by chance alone, this would be indicative of initially transduced hematopoietic progenitor cells. In an extension of the analysis, a certain contamination rate by FACS was to be accounted for.

The statistical inference (expected values E(coinc) of the number of coincidences and p-values p for the observed number of coincidences) is carried out under the null hypothesis H_0_ that, if no contamination occurs, the IS locations in the two cell lines are represented by independent variables with lentiviral distributions as described above. Two situations were considered:

- No contamination.
- Contaminations do occur. It is assumed that the proportion of contaminated cells is the same for both cell types. The analysis takes the robust (worst case) stance that every IS in the contaminated part of the analyzed cells leads to a coincidence.

For the mathematical formulae and some technical details see the Supporting Document Text S4. In case of no contaminations, the formulae and programs (coinc1, coinc2, resp.) permit the exact calculation of E(coinc) and p, whereas, if contaminations are present, only upper bounds can be determined.

## Discussion

Given that nearly all leading gene-therapy studies use integrating viral vectors, there is a need for mathematical and statistical tools tailored for the analysis of viral integration sites. In this paper, we focus on methods and computer programs for the analysis of common integration sites (CIS), with applications both to γ-retroviruses and lentiviruses (which show different integration patterns).

Our methods and programs focus on the analysis of the *number* of CIS. When starting the development of our analytical tools, we decided to use the same methodological framework (see the General Methodology section above) as had been proposed in earlier publications on the subject, an approach which was deemed statistically valid. Meanwhile, alternative definitions and methodologies have been developed, which are more specifically tailored to the challenge of detecting significant clusters of IS. deRidder et al. [27] proposed a different definition of CIS, based on peaks of the (smoothed) density function of the IS. Using the results from computer simulations, the critical peak height for defining a CIS is specified such that the multiple level of significance α is controlled. This method of analysis, which is primarily concerned with the general concept of clustering and does not distinguish between the orders of the CIS, cannot be reproduced by our programs, but may well be used in a complementary way.

Starr et al. [28] adapted the window sizes to the number of IS in the data set under consideration, such that the expected numbers E of CIS (of the order n to be analyzed) under the null hypotheses (i.e. assuming a certain distribution for the IS) is <1. This approach for defining and detecting CIS is within the scope of our methods, although two steps are needed to reproduce them. Note that our formulae or programs are flexible as regards the window sizes for defining CIS, i.e., these sizes can be chosen at the investigator's discretion. The window size leading to E<1 under the null hypothesis (with the given number of IS in the data set) can be determined either by applying the formulae, or, perhaps more appropriately, by means of the simulation programs which take into account the particular distribution of the genes and TSS. This critical window size can then be applied in the programs for detecting and analyzing CIS in the particular data set.

We have devised formula-based approaches useful for a quick analysis, as well as simulation-based methods, which are appropriate for samples showing intensive clustering in specific regions and which take the entire exact genome localization of the TSS (in case of γ-retroviruses) or of genes (in case of lentiviruses) into account.

An overview of the program package is given in Table 1.

**Table 1:** Major constituents of the program package CIS.

Program	Objective
**1. Formula-based methods**	
*cis, cisv*	expected numbers of CIS and p-values for the observed numbers of CIS, assuming a uniform distribution of the IS
*cisretro*	ditto, γ-retroviral IS distribution (CIS of order 2 only)
*cislenti*	ditto, lentiviral IS distribution
*coinc1*	coincidences of IS in two cell types without contaminations
*coinc2*	coincidences of IS in two cell types with contaminations
*compsk*	comparison of the numbers of CIS from two experiments with different numbers of IS (with expected numbers given)
*compsk_retro*	ditto for γ-retroviral IS distribution and unknown expected numbers (only CIS of order 2)
**2. Basic modules used in Monte Carlo methods**	
*ciscount*	counting of CIS in a given set of IS locations
*isinciscount*	counting of IS involved in CIS
*idsincisdet*	enumeration of the locations of IS involved in CIS
*fvis*	generation of uniformly distributed IS locations
*feval*	statistical analysis of the results of simulation studies
*compress*	subroutine used to compress highly disconnected genomic regions produced when discarding the I_TSS_
*ftssc, ftssu*	generation of randomly distributed IS in the I_TSS_
**3. Monte-Carlo methods**	
*cis_simul*	expected numbers and p-values for CIS and IS involved in CIS (expected numbers based on uniform IS distribution, p values based on given total numbers)
*CISUNIFc*	ditto, using given IS locations; conditional analysis
*CISUNIFu*	ditto, using given IS locations; unconditional analysis
*CISRETROc*	ditto, with expected numbers based on a γ-retroviral IS distribution; conditional analysis
*CISRETROu*	ditto, with expected numbers based on a γ-retroviral IS distribution; unconditional analysis
*CISLENTIc*	ditto, with expected numbers based on a lentiviral IS distribution; conditional analysis
*CISLENTIu*	ditto, with expected numbers based on a lentiviral IS distribution; unconditional analysis
*comp1*, *comp2*	comparison of the numbers of CIS from two experiments with different numbers of IS (assuming uniform IS distribution), according to method1 and 2, resp. (see text)

For each IS distribution modeled in the simulations, two different methods of analysis were implemented: a *conditional* one using observed number of IS for the specific genomic regions addressed by the models, and an *unconditional* one based on expected values for these numbers.

In the *conditional* analysis, the number of IS attributed to each chromosome C (under H_0_) is simply equal to the observed number of IS on C. In addition, this analysis is conditional on the model parameters, which means that the observed proportions of IS in I_TSS_ (in case of CISRETROc) or in gene coding regions (in case of CISLENTIc) on each chromosome are used in place of assumed values. By contrast, in the *unconditional* analysis, expected instead of observed numbers of IS are used. Thus, e.g., the number of IS attributed to a chromosome C under H_0_ is calculated from the total number of IS by using weights proportional to the characteristics of C.

While the unconditional analysis is useful for trying and assessing hypothetical models, conditioning, at least on the model parameters, is preferable in the analysis of real data, where estimations of these parameters are available. As for chromosomes, the considerations are different, because (in contrast to the parameters p_TSS_ or q_G_) the proportions of IS on each chromosome are not among the parameters of the mathematical models. An analysis without conditioning on the chromosome essentially treats the chromosomes as undistinguishable, except for characteristics specified in the IS distribution under H_0_, e.g. the locations of gene coding regions or TSS. By contrast, conditioning on the chromosomes is appropriate if there is evidence (either biological or statistical one) that further factors exist - of little or no interest, but differing across the chromosomes - affecting the number of IS and thus (indirectly) the expected number of CIS. In the conditional analysis, these chromosome-specific influences on the number of CIS are corrected for by taking them into account under H_0_, i.e., in the simulated distribution of the IS.

Summarizing, the unconditional and conditional approaches differ in their assumptions, methods of analysis, and results (see *Example 4*). The points raised above may be helpful in deciding which approach is more appropriate in a particular situation.

The comparison of the integration patterns, and in particular CIS, in different clinical gene therapy studies necessitates an adjustment for different numbers of IS. We present two different methods of adjustment: a formula-based approach, which has a theoretical foundation but is sensitive to assumed values of the input parameters, and a simulation-based approach which is less limited in scope and does not have explicit distributional assumptions, but is somewhat heuristic.

Another challenge closely related to CIS analysis is the occurrence of coincidences of IS in different cell types. In many gene therapy studies such coincidences may help to understand which cell-type was initially transduced and how the differentiation occurs. We have developed methods and computer programs comparing the observed number of coincidences with the number to be expected by chance alone, accomodating a certain level of contamination.

In our lab, the presented programs have been applied to various experimental samples and proven helpful in assessing potential vector-induced side-effects.

## Supporting Information

Text S1 **Expected value E(cis_5_) for the CIS of order 5 under a uniform distribution of the IS.** The resulting formula for CIS of order n = 5 is given. We use the notation and terminology introduced in the Methods section of the manuscript.
(DOC)

Text S2 **Expected values E(cis_2_) for γ-retroviral distributions.** Modeling a more general γ-retroviral distribution of the IS allowing for arbitrary halfwidth w of the intervals ITSS, and including a slight correction. We use the notation and terminology introduced in the Methods section of the manuscript.
(DOC)

Text S3 **Technical remarks regarding conditional/unconditional CIS analysis.** Differences between the conditional an unconditional CIS analysis for a) uniform IS distribution, b) lentiviral IS distribution, c) γ-retroviral IS distribution. We use the notation and terminology introduced in the Methods section of the manuscript.
(DOC)

Text S4 **Formulae for the number of coincidences of IS in different cell types.** Two situations were considered: a) No contaminations, b) contaminations do occur. We use the notation and terminology introduced in the Methods section of the manuscript.
(DOC)
