# Supplementary material for: Analyzing the Number of Common Integration Sites of Viral Vectors – New Methods and Computer Programs
Source: PLoS One. 2011 Oct 14;6(10):e24247. doi: 10.1371/journal.pone.0024247 (PMC3194800; doi:10.1371/journal.pone.0024247)
Supplement: Text S1 — Expected value E(cis5) for the CIS of order 5 under a uniform distribution of the IS. The resulting formula for CIS of order n = 5 is given. We use the notation and terminology introduced in the Methods section of the manuscript. (DOC) [file pone.0024247.s001.doc]

By following the approach outlined in Abel et al. [31], and exploiting that, for all i,m,

$$\sum_{i=1}^{m} i^{3} = \frac{m^{2}(m+1)^{2}}{4}$$

one obtains

(1) $E(cis_{5})\approx\left(\begin{matrix} is \\ 5 \end{matrix}\right)\frac{1+5(d_{5}+1)d_{5}[d_{5}(d_{5}+1)+1]}{g^{4}}$
