# Supplementary material for: Analyzing the Number of Common Integration Sites of Viral Vectors – New Methods and Computer Programs
Source: PLoS One. 2011 Oct 14;6(10):e24247. doi: 10.1371/journal.pone.0024247 (PMC3194800; doi:10.1371/journal.pone.0024247)
Supplement: Text S2 — Expected values E(cis2) for γ-retroviral distributions. Modeling a more general γ-retroviral distribution of the IS allowing for arbitrary halfwidth w of the intervals ITSS, and including a slight correction. We use the notation and terminology introduced in the Methods section of the manuscript. (DOC) [file pone.0024247.s002.doc]

The following formulae (2a-2e) are similar to those given in Abel et al. [31], but allowing for a variable half-width w of the I_TSS_, and including the parameter p_TSS_. In addition, formula (2e) contains a correction to the corresponding formulae (13, 14) in Abel et al. [31].

We assume a uniform IS distribution in the complement of the I_TSS_. If w≤d_2_/2 and p_pref_=1, then, if CIS formed by IS located in different I_TSS_ are neglected, we have:

(2a) $E(cis_{2}) =E_{TSS}+E_{mix}+E_{Comp}$

where E_TSS_,E_mix_,E_Comp_ denote the expected values of the number of CIS of order 2 formed from IS in I_TSS_ only, from one IS in an I_TSS_ and one in the complement, and from two IS lying in the complement of the I_TSS_, respectively.

(2b) $E_{TSS}\approx\left(\begin{matrix} is\cdot p_{TSS} \\ 2 \end{matrix}\right)/n_{TSS}$

(2c) $E_{Comp}\approx\left(\begin{matrix} is\cdot(1-p_{TSS}) \\ 2 \end{matrix}\right)\frac{2d_{2}+1}{(g-2n_{TSS}w)}$

(2d) $E_{mix} = is^{2}p_{TSS}(1-p_{TSS})p_{mix}$

where p_mix_ denotes the probability that any randomly chosen pair of IS with one element in a given I_TSS_ and one element in the complement forms a CIS of order 2:

(2e) $p_{mix } \approx\frac{2(d_{2}-w)}{(g-2n_{TSS}w)}$

If p_pref_≠1 then in formulas (2b-e) n_TSS_ merely has to be replaced with p_pref_⋅n_TSS_.

(2b) and (2e) do not depend on any assumption regarding the distribution on IS in the I_TSS_. In both cases, this follows from the assumption w≤d_2_/2. For (2b) this is obvious; for (2e) the **proof** is as follows:

For a given x inside of an I_TSS_ I, let I_r_(x),I_l_(x) be the intervals to the right and left of I such that every y in I_r_(x)∪I_l_(x) forms a CIS of order 2 with x. Because of w≤d_2_/2 both intervals are nonempty, and their total length is 2(d_2_-w), regardless of the particular position of x in I. This follows from the fact that I_r_(x)∪I_l_(x) is simply the set of all IS having distance ≤d_2_ from x, with I cut out from this set. From our assumption that the TSS are sufficiently sparse or, more precisely, that CIS from IS lying in two different I_TSS_ can be neglected, it follows that y may be assumed to be located in the complement of all I_TSS_, implying that the IS are uniformly distributed in I_r_(x)∪I_l_(x). Hence, the probability for a particular y (known not be located in an I_TSS_) to belong to the particular set I_r_(x)∪I_l_(x) is given by the length of this set relative to the total complement of the I_TSS_, i.e. the right side of (2e). Since this result is independent of the location of x in I, it is also independent of the spatial distribution of the IS in I.
