# Supplementary material for: Analyzing the Number of Common Integration Sites of Viral Vectors – New Methods and Computer Programs
Source: PLoS One. 2011 Oct 14;6(10):e24247. doi: 10.1371/journal.pone.0024247 (PMC3194800; doi:10.1371/journal.pone.0024247)
Supplement: Text S3 — Technical remarks regarding conditional/unconditional CIS analysis. Differences between the conditional an unconditional CIS analysis for a) uniform IS distribution, b) lentiviral IS distribution, c) γ-retroviral IS distribution. We use the notation and terminology introduced in the Methods section of the manuscript. (DOC) [file pone.0024247.s003.doc]

The following explanations may highlight the differences between the two approaches.

a) Uniform IS distribution.

In the unconditional analysis assuming a uniform distribution of the IS, the expected number of IS allocated to a chromosome C in the computer simulations is simply given by

is·g_C_/g,

where g,g_C_ are the length of the genome and of the gene-coding region on C, respectively. By contrast, the conditional analysis uses the actual number of IS observed on C.

b) Lentiviral IS distribution.

In the unconditional analysis the expected number of IS on gene coding regions G and their complement H (and thus the basis for the number of IS allocated to these regions in the computer simulations) are given by is·q_G_ and is·q_H_, respectively, while the expected number of IS on chromosome C is given by

is·(q_G_·g_G,C_/g_G_ + q_H_·g_H,C_/g_H_),

where g_G_,g_H_,g_G,C_,g_H,C_ are the total length of gene coding regions and their complement in the genome and on C, respectively. CISLENTIu provides goodness-of-fit statistics for the chromosomes corresponding to this unconditional model. By contrast, in the conditional analysis the numbers of IS actually observed in the gene coding regions and their complement on each chromosome are used in the computer simulations.

c) γ-retroviral IS distribution.

The unconditional analysis is quite analogous to that for lentiviruses, here p_TSS_ being the model parameter in question.

Note that the programs allow for conditioning on the model parameters without conditioning on chromosomes, namely, by using the unconditional version with the observed values of the model parameters in place of the assumed ones.

It is also noteworthy that for all models considered here, if H_0_ is satisfied for each chromosome separately, it is also satisfied for the total genome; i.e., conditioning on chromosomes preserves the model structure.
