# Supplementary material for: Analyzing the Number of Common Integration Sites of Viral Vectors – New Methods and Computer Programs
Source: PLoS One. 2011 Oct 14;6(10):e24247. doi: 10.1371/journal.pone.0024247 (PMC3194800; doi:10.1371/journal.pone.0024247)
Supplement: Text S4 — Formulae for the number of coincidences of IS in different cell types. Two situations were considered: a) No contaminations, b) contaminations do occur. We use the notation and terminology introduced in the Methods section of the manuscript. (DOC) [file pone.0024247.s004.doc]

a) No contaminations: We give the relevant formula (see Cartier et al. ) in a slightly more general form which accommodates differing, i.e., cell-specific, proportions of integrations into gene-coding regions:

(3) $E \approx(2d+1)is_{1}is_{2}\left\{\left.\frac{q_{G,1}q_{G,2}}{g_{G}} + \frac{(1-q_{G,1})(1-q_{G,2})}{g-g_{G}}\right\}\right.$,

where d is maximal distance [base pairs] allowed for pronouncing identity or coincidence of two vector integrations; is_1_,is_2_=number of vector integration sites (IS) investigated in cell type 1 and 2, resp.; g= total length of the human genome; g_G_=total length of the gene coding regions; q_G,1_,q_G,2_=true proportions of the vector integration sites located in the gene coding regions. While for calculating scenarios hypothetical values of q_G,1_,q_G,2_ may be assumed, the use of the observed estimators for q_G,1_,q_G,2_ is usually advisable when concrete data are analysed.

Using formula (3) and assuming that the number c of observed coincidences has a Poisson distribution with λ=E, p-values can be calculated.

b) The case of contaminations. We present a simplified, robust approach with few assumptions. Let p_cont_ be the proportion of contaminated cells. We assume that p_cont_ is the same for both cell types. Also we assume the worst case that every IS in the contaminated part of the analyzed cells leads to a coincidence.

First, obviously

E= E_cont_ +E_nocont_ ≤ E_cont_ +E_nocont,max_

where E_cont_ and E_nocont_ denote the expected number of coincidences in which contaminations are involved/not involved, respectively, and E_nocont,max_ is calculated as in formula (3), i.e. assuming that no contamination exists.

For E_cont_ we have

E_cont_ =E'_cont_ + E''_cont_ .

E'_cont_ represents coincidences composed of IS in (i) cells of type 1 cells contaminating cells of type 2 and (ii) cells of type 1 identified, plus (iii) and (iv), the analogous number with cell types reversed. E''_cont_ represents coincidences resulting from contaminations only, i.e., coincidences of an IS in a lymphoid cell contaminating the myeloid cells with an IS in a myeloid cell contaminating the lymphoid cells. Clearly, E’’_cont_ is mostly extremely small.

We derive expressions for E'_cont_ and E''_cont_ based on pcont. Observe that p_cont_ refers to *cells*, not IS. If the proportions of lymphoid and myeloid cells having IS are identical (p_is,1_=p_is,2_) then we have

E'_cont_=p_cont_(is_1_+is_2_)

and

E''_cont_=E* p^2^_cont_

where E is calculated using formula (3). Summarizing, this leads to

$$\begin{matrix} (4) E\leq(2d+1)is_{1}is_{2}\left\{\left.\frac{q_{G,1}q_{G,2}}{g_{G}} + \frac{(1-q_{G,1})(1-q_{G,2})}{g-g_{G}}\right\}\right.(1+p^{2}_{cont}) + p_{cont}(is_{1}+is_{2}) \\ \end{matrix}$$

If p_is,1_ ≠p_is,2_, the proportions p_is,1_,p_is,2_ and the numbers of examined cells need to be taken into account. Note that because of the contamination these proportions are only approximately known. Let n_1_,n_2_ be the number of examined cells which are thought to be lymphoid and myeloid, respectively. Then

$$E'_{cont} = p_{cont}(n_{1}p_{is,2} + n_{2}p_{is,1})$$

The two terms on the right side are the expected values of the number of IS in the two contaminated parts. As for E''_cont_, it is still calculated using formula (3), but now is_1_ and is_2_ have to be replaced with p_cont_ ⋅n_l_ ⋅p_is,2_ and p_cont_ ⋅n_2_⋅p_is,l_ , respectively.

For the statistical analysis it is assumed that the number of coincidences follows a Poisson distribution with parameter E. An upper bound for the p-value is then obtained if the parameter E is replaced by the upper bound E_nocont,max_+E'_cont_+E''_cont_, as specified above.

The analysis was implemented in the R programs *coinc1(c,is_1_,is_2_,d,g,g_G_,q_G,1_,q_Ge,2_,p_cont_*) and *coinc2(c,is_1_,is_2_,n_1_,n_2_,d,g,g_G_,q_G,1_,q_Ge,2_,p_cont_,p_is,1_,p_is,2_*) pertaining to the cases of no contaminations/ contamination, respectively

The specification of is_1_,is_2_ in the program coinc2 might appear unnecessary given that

is_1_ ≈ n_1_ {(1-p_cont_) p_is,1_ + p_cont_ p_is,2_},

the right side being the expected value of is1. (An analogous relationship holds for is2). However, we preferred not to rely on approximations where observed values are available.
